# Existing Infection Facilitates Establishment and Density of Malaria Parasites in Their Mosquito Vector

**DOI:** 10.1371/journal.ppat.1005003

**Published:** 2015-07-16

**Authors:** Laura C. Pollitt, Joshua T. Bram, Simon Blanford, Matthew J. Jones, Andrew F. Read

**Affiliations:** 1 Centre for Immunity, Infection and Evolution, University of Edinburgh, Edinburgh, United Kingdom; 2 Center for Infectious Disease Dynamics, Departments of Biology and Entomology, Pennsylvania State University, State College, Pennsylvania, United States of America; 3 Fogarty International Center, National Institutes of Health, Bethesda, Maryland, United States of America; Institut Pasteur, FRANCE

## Abstract

Very little is known about how vector-borne pathogens interact within their vector and how this impacts transmission. Here we show that mosquitoes can accumulate mixed strain malaria infections after feeding on multiple hosts. We found that parasites have a greater chance of establishing and reach higher densities if another strain is already present in a mosquito. Mixed infections contained more parasites but these larger populations did not have a detectable impact on vector survival. Together these results suggest that mosquitoes taking multiple infective bites may disproportionally contribute to malaria transmission. This will increase rates of mixed infections in vertebrate hosts, with implications for the evolution of parasite virulence and the spread of drug-resistant strains. Moreover, control measures that reduce parasite prevalence in vertebrate hosts will reduce the likelihood of mosquitoes taking multiple infective feeds, and thus disproportionally reduce transmission. More generally, our study shows that the types of strain interactions detected in vertebrate hosts cannot necessarily be extrapolated to vectors.

## Author Summary

Very little is known about how malaria parasite strains interact with each other inside mosquitoes. In this study we show that mosquitoes that have already been infected with one strain of malaria parasites are more likely to become infected with a new strain. Moreover, the presence of an existing infection enhances the replication of malaria parasites with no obvious impact on mosquito survival. Our results illustrate that interactions between strains are important factors in parasite survival and transmission across the whole of their life cycle.

## Introduction

Interactions between pathogen strains within hosts can be profound and affect many aspects of infectious disease biology, including disease severity and infectiousness, as well as the evolution of virulence and the spread of drug resistance [1–7]. Yet for medically important vector-borne diseases, very little is known about the nature and implications of strain interactions within the vector. This is in striking contrast to what is known about strain interactions in the vertebrate host.

For example, malaria parasites in mixed strain infections experience significant competitive suppression within the vertebrate host [8–17]. Whether competitive suppression also occurs in their mosquito host is unknown. The progression through the vector is relatively long and complex [18] and involves severe population bottlenecks [19]. Parasite density also influences both the development of the parasite and the probability of the vector surviving for long enough to infect a new host [20–22]. Therefore, strain interactions that increase or decrease parasite density are likely to alter the probability of transmission to a new vertebrate host.

Mixed strain (genotype) infections in mosquitoes are common [23,24] and there are three distinct but non-exclusive routes by which they could arise. First, multiple parasite strains could be taken up from a host during a single blood meal. Mixed strain infections are the norm in areas of high transmission [25], and multiple parasite strains can be transmitted to a vector from a single infective feed [26]. Second, mosquitoes that are disturbed during feeding may move to a new host, resulting in multiple hosts contributing blood to one feeding cycle [27–29]. Finally, mosquitoes could feed on different hosts in successive blood feeding cycles. Studies on human and bird malaria parasites have suggested that mosquitoes that take multiple infective feeds have higher oocyst burdens and parasites at different stages of development, which is suggestive of the accumulation of infections over multiple feeding cycles [30–33]. What impact this has on parasite development or on vector survival not been previously tested. If secondary infections are equally likely to be acquired, then of the mosquitoes surviving to become infectious, up to ~40% of infectious mosquitoes could have oocysts, and up to ~17% could have sporozoites originating from multiple feeds (Fig A in S1 Text). The possibility that mosquitoes can acquire mixed infections from multiple feeds is interesting in its own right, but experimentally, infection from successive blood meals would also provide a way to analyse the competitive interactions between strains without the confounding problems of strain recombination. Parasites in the same blood meal freely recombine in the mosquito gut. There can be no recombination between strains acquired in different feeding cycles because zygotes are formed within a few minutes of a blood meal. When a successive meal takes place several days later, all gametes from the first meal are gone [34].

Here we show that mosquitoes can accumulate mixed strain infections from feeding on multiple hosts, and that the presence of oocysts from an existing parasite infection make subsequent infections more likely and more productive. Additionally, we show that vector mortality was no higher for double infections than for infections with a single parasite strain.

## Results

### Mosquitoes can accumulate infections from multiple feeds

An initial study (experiment 1) was conducted to test whether it is possible for mosquitoes to pick up multiple infections from multiple bloodmeals. Six cages, each containing ~100 three to five day old *Anopheles stephensi* female mosquitoes were used. Half of the cages fed on mice infected with the rodent malaria parasite *Plasmodium chabaudi* (strain ER), and half received an uninfected blood meal (control). Four days after their initial feed, all cages of mosquitoes received a second blood meal containing *P*. *chabaudi* strain AJ parasites. This 4 day schedule corresponds to the preferred blood-feeding frequency for female mosquitoes [35–37]. Seven days after the second blood meal (experimental day 11) when parasites from the second feed were expected to have established as mature oocysts, ~30 mosquitoes per cage were removed, dissected and tested for the presence and density of each of the parasite strains by genotype specific PCR on infected midguts (see Table 1 for treatment groups and sample sizes). A comparison with mosquitoes dissected four days earlier confirmed that our ability to detect infections from the first feed did not decline over this time period (S1 Fig).

**Table 1:** Treatment groups and sample sizes.

	1^st^ feed	2^nd^ feed	n (mosquitoes*, cages)	Dissected day 7	Dissected day 11
**Experiment 1**					
	Control	AJ	338, 3	60	92
	ER	AJ	278, 3	80	77
**Experiment 2**					
	Control	Control	232, 3	-	-
	Control	AJ	212, 3	-	90
	Control	ER	195, 3	-	86
	AJ	Control	222, 3	91	-
	AJ	ER	294, 3	90	88
	ER	Control	202, 3	90	-
	ER	AJ	256, 3	90	91

* Mosquitoes which took full blood meals on both transmissions. Control = bloodmeal on an uninfected age and sex matched mice.

**Fig 1:**
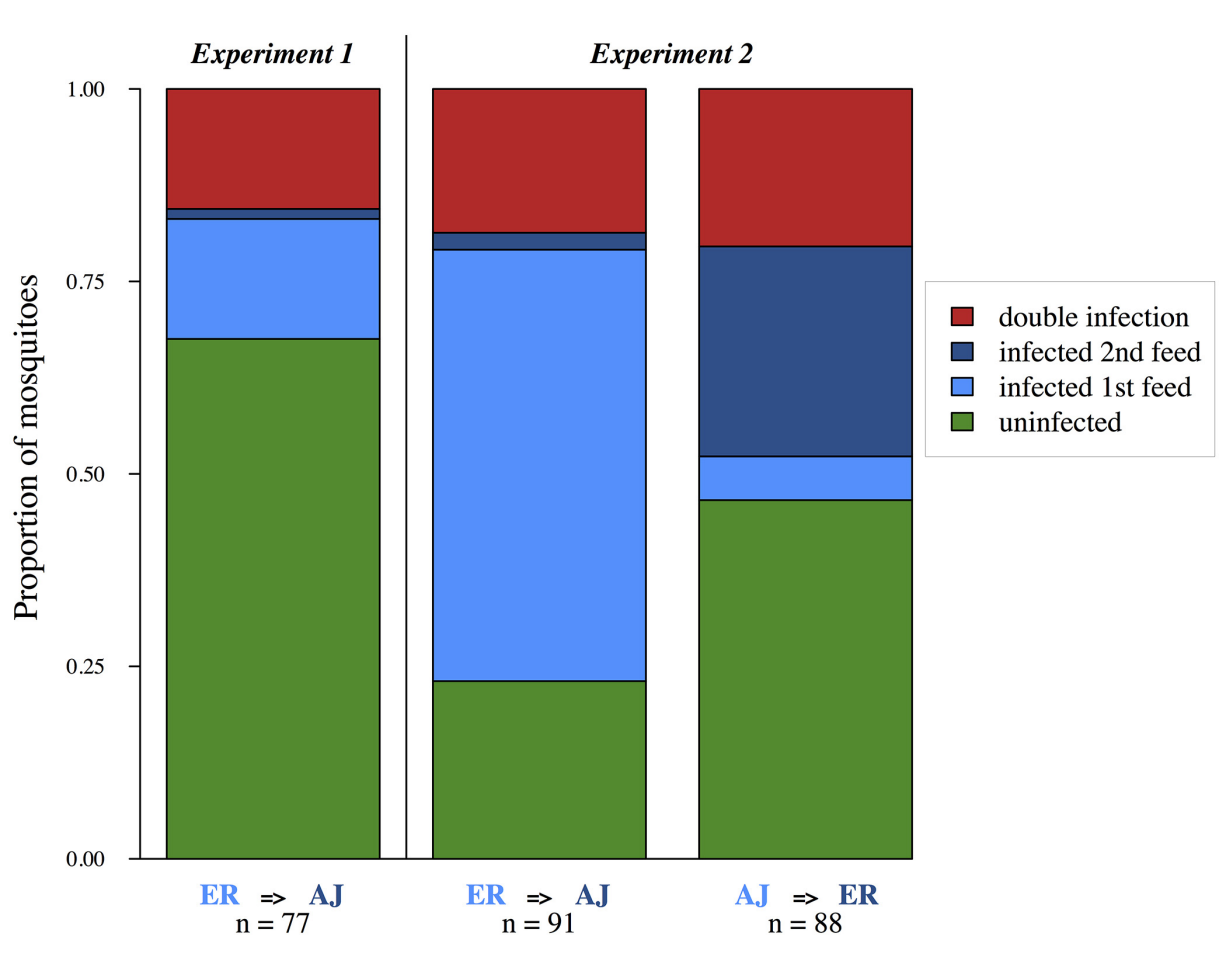
Mosquitoes can accumulate multiple infections from successive bloodmeals. Each bar shows pooled data for mosquitoes from 3 experimental replicates (cages). Presence of each genotype determined by PCR of infected midguts 7 days after the second bloodmeal (experimental day 11). Treatment group (first feed = > second feed) and sample sizes are shown below each bar.

We found that mosquitoes become doubly infected with parasites from successive blood meals. A total of 31%(±5.3 SEM) of mosquitoes became infected with ER parasites during their first feed and of these infected mosquitoes 50%(±10.4 SEM) additionally became infected with AJ parasites during their second feed (Fig 1).

We then conducted a second larger study (experiment 2) with 21 cages, again each containing ~100 female mosquitoes. Six cages received two infective blood meals with one each of our two parasite strains (3 x AJ-ER and 3 x ER-AJ), six cages received an infective blood meal only on their first feed (3 x AJ-C and 3 x ER-C), six cages received an infective blood meal only on their second feed (3 x C-AJ and 3 x C-ER), and finally three cages received two uninfected blood-meals (C-C) (Table 1). All cages received two blood meals with mosquitoes in single infection treatments being given an uninfected feed in place of one of the infective blood meals. This was done in order to control for any effect of a second blood meal on parasite replication [38]. This fully factorial study design allowed us to examine how the presence of a co-infecting strain affects parasites that enter the vector first and second, and to test whether co-infection impacts vector survival.

The six cages that received two infective feeds were all found to contain mosquitoes infected with parasites of both strains. For cages which fed on ER first and AJ second, 75%(±4.6 SEM) of mosquitoes became infected on their first feed, and of these 25%(±5.3 SEM) additionally became infected with AJ. For cages that fed on AJ first and ER second, 25%(±4.7 SEM) of mosquitoes became infected on their first feed, and of these 78%(±8.8 SEM) additionally became infected with ER (Fig 1). Therefore both parasite strains were able to establish in already infected vectors.

### The affect of secondary infection on replication of primary infection

**Fig 2:**
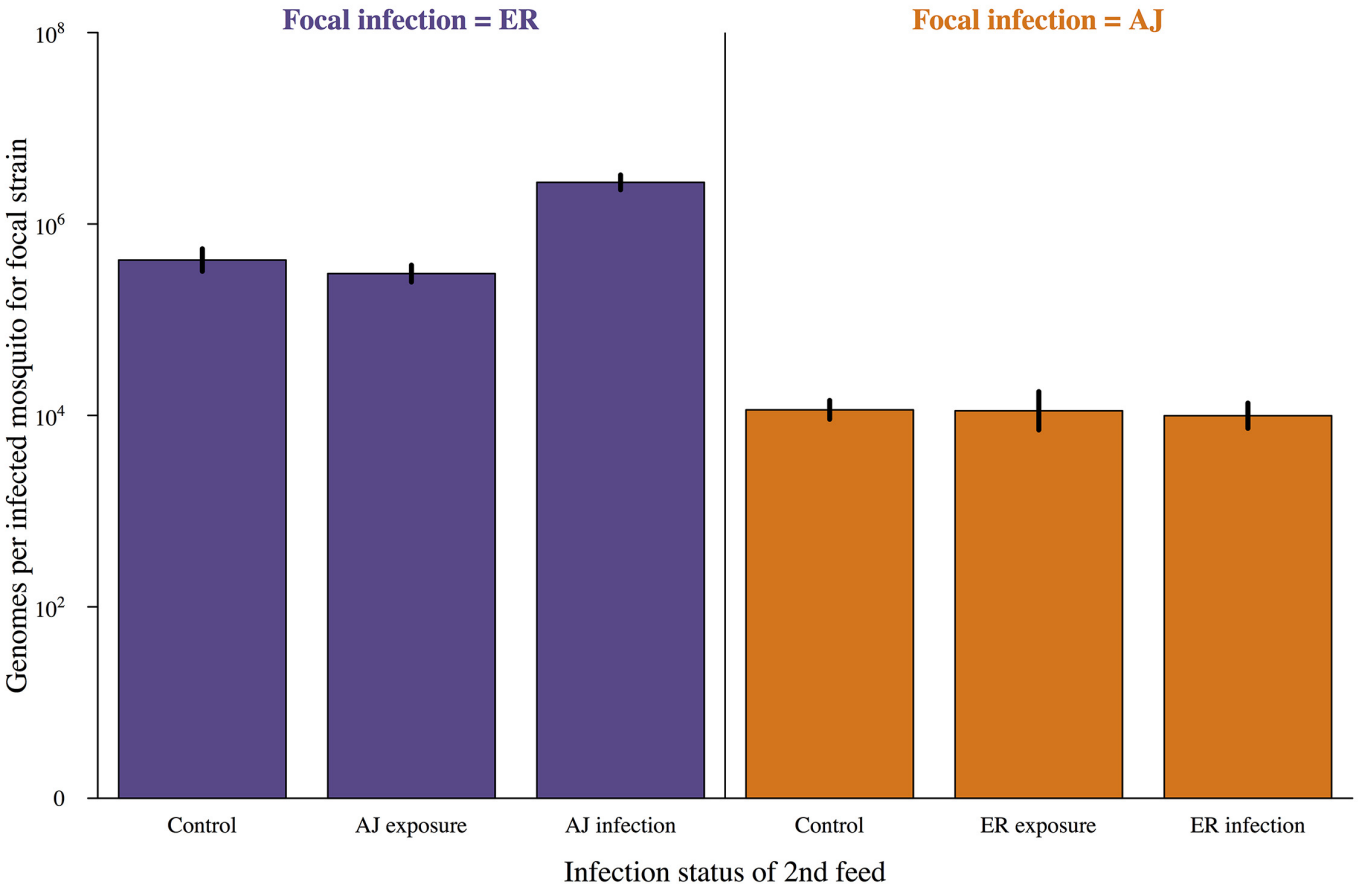
Subsequent infection does not negatively impact on parasite development. Genome count (number of potential sporozoites produced) for infections established after a mosquitoes first blood meal depending on the status of the second feed received. Control = second bloodmeal from an uninfected host, exposure = second bloodmeal from an infected host but second infection did not establish, infection = secondary infection established. Mosquitoes were dissected and genome numbers and the presence of secondary infection determined by PCR 7 days after their first bloodmeal. Means calculated from 90–100 mosquitoes across 3 cages per combination and bars show the standard error of the mean. Genome density was significantly affected by focal parasite strain (AJ vs. ER; χ^2^ = 21.13, p<0.001) but not by treatment group (control vs. infective 2^nd^ feed; χ^2^ = 0.09, p = 0.77). For full details of analyses see results text and Table B in S2 Text.

It was not possible to determine which feed individual oocysts originated from, but by using quantitative PCR we were able to determine the genome count (total number of potential sporozoites produced) for each of our strains within each infected mosquito midgut. The production of sporozoites within the oocyst requires the acquisition of (presumably limited) nutrients from the mosquito [27,39] and has previously been shown to be negatively related to oocyst density [20]. Due to anaemia and immune factors from the vertebrate host, infective bloodmeals are also likely to be lower quality. Therefore, we predicted that the host infection status and/or the establishment of a new infection during oocyst development would negatively impact parasite replication (competitive suppression). However, the host infection status of the second bloodmeal (infective or control) did not affect the number of genomes from the first infection for either of our focal strains (Treatment (infective or control): χ^2^ = 0.01, p = 0.77; Treatment*Focal strain: χ^2^ = 0.20,p = 0.66; Fig 2; Table B in S2 Text). When we split our infective treatment group by whether the second infection established or not, we found no effect of secondary infection on AJ (Control vs. Infected: z = 0.24, p = 0.99; Fig 2; Table B in S2 Text), and for ER infections genome numbers were actually slightly higher in mosquitoes which were subsequently infected with AJ (Control vs. Infected: z = 3.49, p = 0.01; Fig 2; Table B in S2 Text). This suggests that the development of established malaria infections is not negatively impacted by secondary infections.

### The effect of previous infection on probability of secondary infection

**Fig 3:**
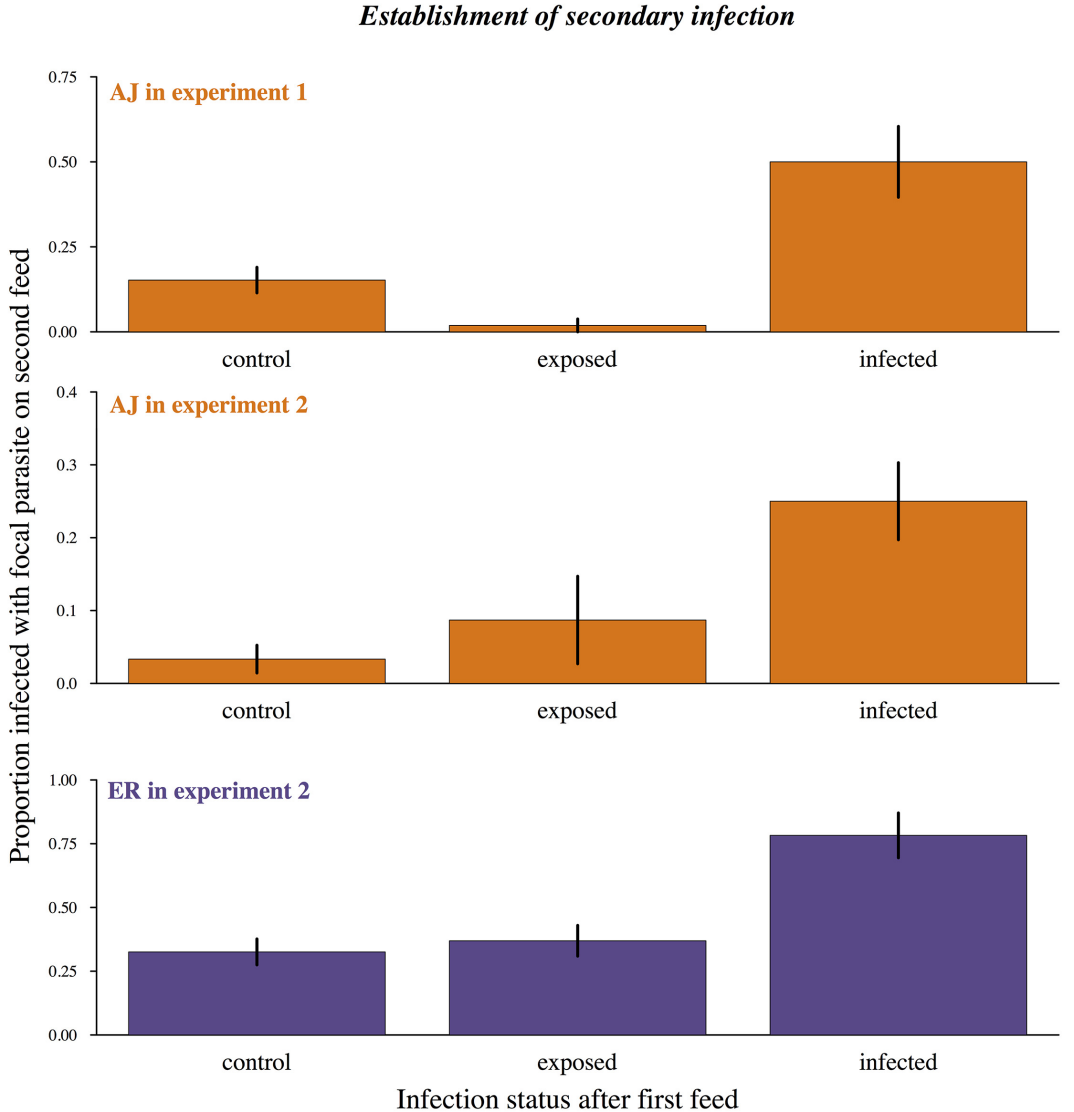
Already infected mosquitoes are more likely to pick up a second infection. Proportion of mosquitoes becoming infected during their second bloodmeal depending on previous infection status. Control = first bloodmeal from an uninfected host. Exposed = first bloodmeal infective but primary infection did not establish. Infected = mosquito already infected from first bloodmeal at the time of second bloodmeal. Means calculated from 86–95 mosquitoes across 3 cages per combination and bars show the standard error of the mean. Uninfected control mosquitoes and uninfected but exposed mosquitoes were equally likely to become infected during their second bloodmeal (experiment 1: X^2^ = 2.04, p = 0.1; experiment 2: X^2^ = 2.05, p = 0.15) while mosquitoes with an established infection were significantly more likely to become infected (experiment 1: X^2^ = 21.38, p<0.0001; experiment 2: X^2^ = 7.09, p = 0.008). In experiment 2 there was also a significant effect of focal strain (X^2^ = 7.83, p = 0.005) but no interaction between strain and infection status (X^2^ = 0.44, p = 0.8). For full analysis see results text and Table C in S2 Text.

In our first experiment, AJ was used as our focal strain and was more than five times as likely to infect mosquitoes already infected with ER than mosquitoes which had previously received a control feed or had been exposed to ER on the first feed but had not become infected (previous infection status Χ^2^ = 21.38, p<0.0001; Fig 3; Table C in S2 Text).

In our second experiment, we measured how previous infection affected the establishment of parasites received during the second bloodmeal for both our strains. In agreement with experiment 1, mosquitoes which had become infected during their first feed were much more likely to then become infected on their second feed (infection with focal strain ~ previous infection status Χ^2^ = 7.09, p<0.01; Fig 3; Table C in S2 Text).

Infection probabilities varied with focal strain and experiment (Fig 3), which was likely due to mice having lower gametocyte densities for AJ infections in experiment 2 (Table A in S2 Text). However, the relative increase in infection probability during a second feed for previously infected mosquitoes remained consistent (previous infection status*focal parasite strain in experiment 2: Χ^2^ = 0.44, p = 0.80; previous infection status*experiment for AJ: Χ^2^_2,7_ = 0.99, p = 0.32). Therefore the presence of parasites from a previous infection increased the probability of a new infection for both our focal strains and in replicate experiments.

The observed increase in infection probability during the second bloodmeal for mosquitoes infected during the first could be due to (1) mosquito variation in susceptibility, so that some individuals had a higher likelihood of infection during both feeds, (2) blood-meal quality of the first feed having knock on effects for the second feed (for example, feeding on an anaemic mouse for the first blood-meal could result in mosquitoes taking a larger second blood-meal), or (3) the first infection facilitating the establishment of the secondary infection (either through physical damage to the midgut, changes in resource availability, or immune depletion).

In each of our experiments, mosquitoes where randomly allocated to experimental cages from the same cohort of inbred mosquitoes. It is therefore unlikely that there would be variation in susceptibility between cages, although it is possible that there could be variation in susceptibility between mosquitoes within cages. If there were a subset of mosquitoes refractory to infection in each cage we would expect i) the total number of mosquitoes in each cage to remain constant ii) mosquitoes which failed to become infected during their first feed would be less likely than controls to become infected during a second feed. In both our experiments, cages which received two infectious feeds had an overall higher prevalence of infection from the second feed than in control cages (Χ^2^_1,4_ = 6.07, p = 0.034), suggesting the increase in susceptibility in these cages was occurring over and beyond the background level of infection. Additionally, previously exposed but uninfected mosquitoes were just as likely to become infected on their second bloodmeal as mosquitoes from control cages (Experiment 1: X^2^ = 2.04, p = 0.1; Experiment 2: X^2^ = 2.05, p = 0.2; Fig 3; Table C in S2 Text) and therefore did not represent a refractory subset of individuals.

Differences in blood-meal quality *per se* are also unlikely to explain increased transmission to already infected mosquitoes: mosquitoes that had previously received a control feed or had received an infective feed but remained uninfected were equally likely to become infected during their second bloodmeal (Control vs. exposed: Experiment 1: X^2^ = 2.04, p = 0.1; Experiment 2: X^2^ = 2.05, p = 0.2; Fig 3), and there was no effect of the mouse red blood cell density on probability of infection (Experiment 1: Χ^2^ = 0.01, p = 0.99; Experiment 2: Χ^2^ = 0.10, p = 0.75).

By a process of elimination, it seems most likely that the presence of a primary infection directly increases the chance of a secondary infection establishing. In order to determine how this occurs (e.g. whether through interactions with vector immunity, resources, or physical damage to the mosquito midgut) more experiments are needed.

### The effect of a previous infection on replication of subsequent infection

**Fig 4:**
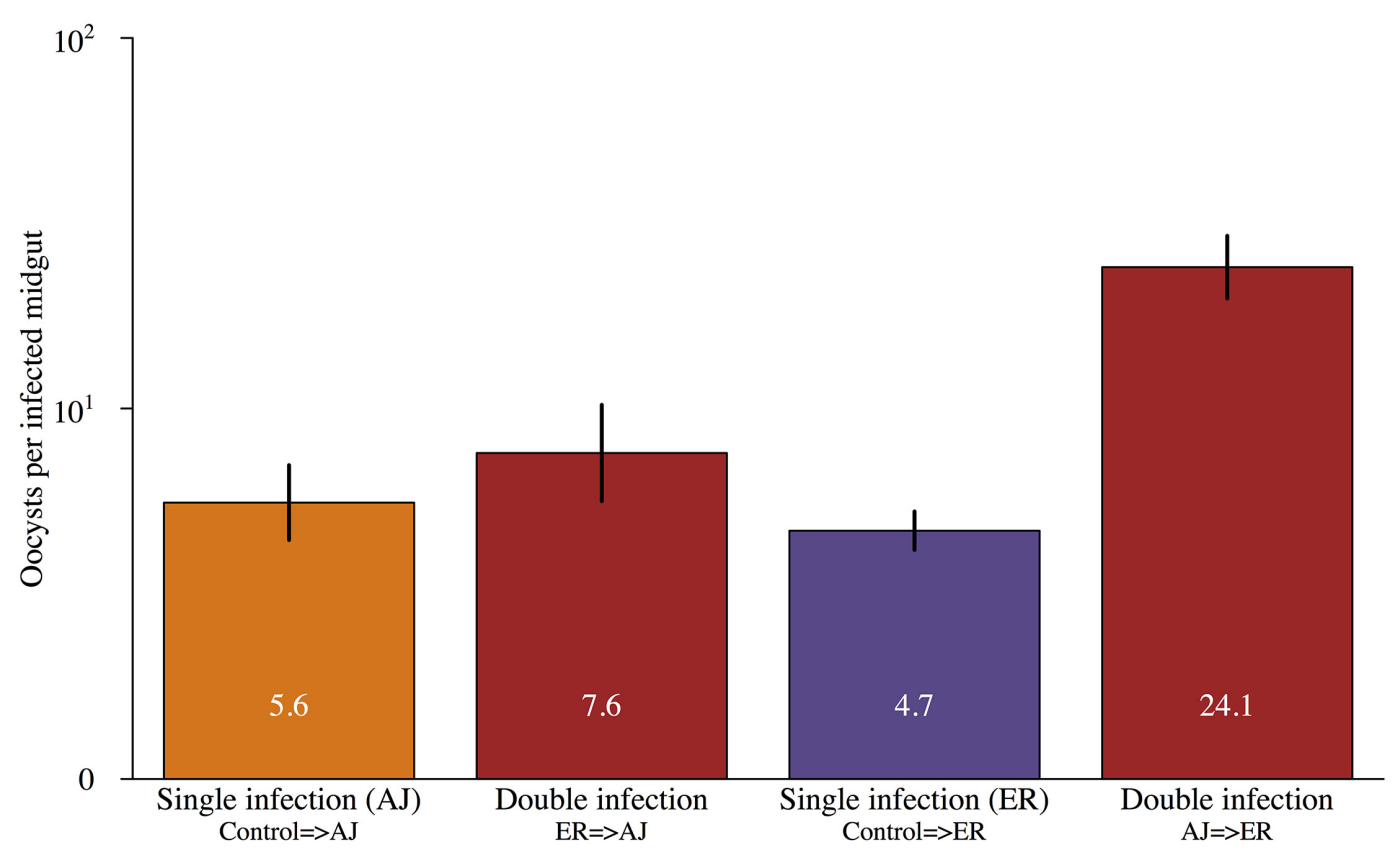
Oocyst loads in mosquitoes infected with parasites from single and multiple feeds. Mean number of oocysts in infected mosquitoes that were only infected on their second feed (single infection) or were infected during both blood meals (double infection). Infection order is shown below bars for double infections e.g. ER = >AJ were infected with ER during their first feed and then AJ on their second. Values within the bars show the mean number of oocysts for each group and error bars show the standard error of the mean. There was a significant interaction between infection status (single vs. double) and focal strain (X^2^ = 60.1, p<0.001) with AJ = >ER double infections significantly higher densities than either ER = >AJ double infections or single infections with either strain. For full details of analyses see results text and Table D in S2 Text.

As expected, overall oocyst burdens were higher in mosquitoes that were infected during both bloodmeals compared to mosquitoes infected only on their second bloodmeal. However, the magnitude of this effect depended on the order of strains in the double infections. The highest oocyst burdens were found in mosquitoes with AJ infections followed by ER infections (oocyst density ~ infection status*focal parasite: X^2^ = 9.22, p<0.005; Fig 4; Table D in S2 Text).

**Fig 5:**
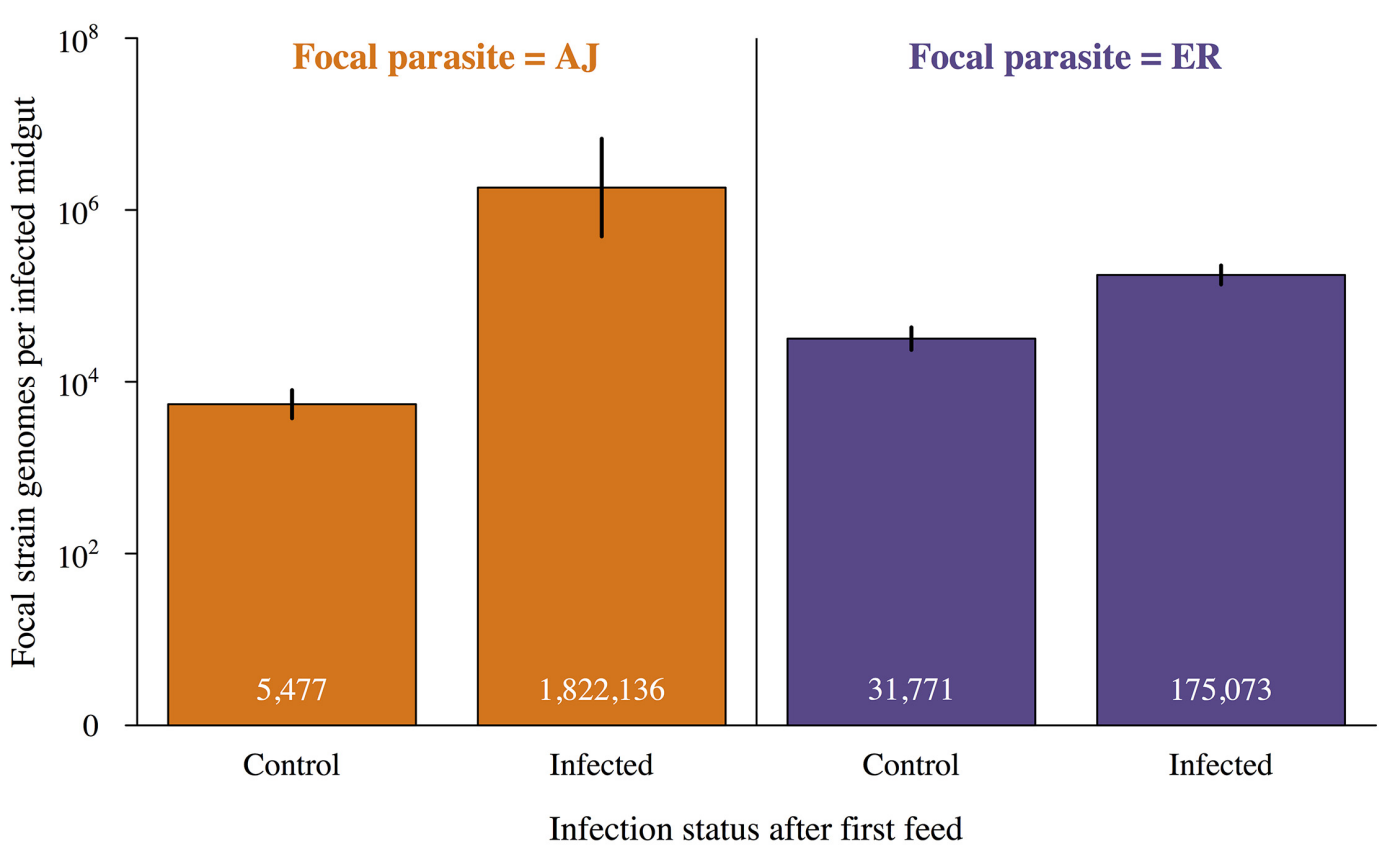
Infections established in already infected mosquitoes have higher genome counts. Mean genome counts per infected mosquito for focal infections established during the mosquitoes second feed depending on whether the mosquito had an established infection from its first feed or had previously received a control (uninfected) bloodmeal. Values within the bars show the mean number of oocysts for each group and error bars show the standard error of the mean. Focal infections in already infected mosquitoes had significantly higher genome counts than focal infections in control mosquitoes (X^2^ = 8.15, p<0.005). Identity of the focal strain did not significantly impact genome count (X^2^ = 0.13, p = 0.72). For full details of analyses see results text and Table E in S2 Text.

It was not possible to reliably determine which infection individual oocysts resulted from, but we were able to compare genome counts for our focal infections developing in double infections those in matched single infections (controls). Infections that established in already infected mosquitoes had higher genome counts than those that established in previously uninfected (naïve) mosquitoes (X_2_ = 8.15, p<0.005; Fig 5; Table E in S2 Text). The magnitude of this effect depended on the focal strain (genome count 6 x higher for ER but over 300 x higher for AJ; Fig 5).

Higher genome counts in already infected mosquitoes could have been due to some mosquitoes being more susceptible to both infections, but genome counts from the first and second infections for double infected mosquitoes were unrelated (Χ^2^_1,8_ = 0.002, p = 0.97; S2 Fig). Therefore, the presence of parasites from a prior infection increases both the chances that subsequent infection will establish, and the density that subsequent infection will reach in the mosquito.

### The effect of infection status on vector survival

The probability that parasites will be transmitted to a new vertebrate host depends both on the ability of the parasite to establish and replicate within the vector and the potential number of infective bites a vector can take, which will depend on how many blood feeding cycles the mosquito survives for. We performed a comprehensive examination of the impact of infection status on vector survival. A total of 1631 mosquitoes across 21 cages were monitored twice daily until death (our longest lived mosquito died 72 days after receiving its first bloodmeal). Three cages fed on uninfected mice during both blood meals (C-C), 12 cages fed on control mice for one bloodmeal and infective mice for the other (C-AJ, C-ER, AJ-C or ER-C), and 6 cages fed on infective mice during both bloodmeals (AJ-ER or ER-AJ) (Table 1). Dead mosquitoes were tested for the presence of infection and identity of the infecting strain(s) using PCR.

There was no significant difference in survival between control uninfected mosquitoes and exposed but uninfected mosquitoes (Χ^2^_1,615_ = 0.003, p = 0.96), therefore these groups were analysed together giving us 4 groups for comparison (uninfected; infected with AJ; infected with ER; infected with both strains). While PCR of mosquito cadavers allowed us to directly determine infection status (uninfected, infected with AJ, infected with ER, or double infection) for mosquitoes used in survival analysis oocyst counts from dead mosquitoes are not possible. Therefore, a mean oocyst density was calculated from a subset of ~30 mosquitoes per cage which were removed and dissected 7 days after each infective bloodmeal. Dissected mosquitoes were counted as censored points in the survival analysis. Total gametocyte densities were taken as the summed gametocyte density from the two feeds taken by each mosquito and red blood cell density was the mean of the two feeds. Across all groups there were no significant relationships between mosquito survival and red blood cell density in the blood-meals (Χ^2^ = 0.001, p = 0.97), mean oocyst density (Χ^2^ = 0.84, p = 0.36), or gametocyte density (Χ^2^ = 3.04, p = 0.08), therefore these factors were dropped from the statistical models (Table F in S2 Text).

**Fig 6:**
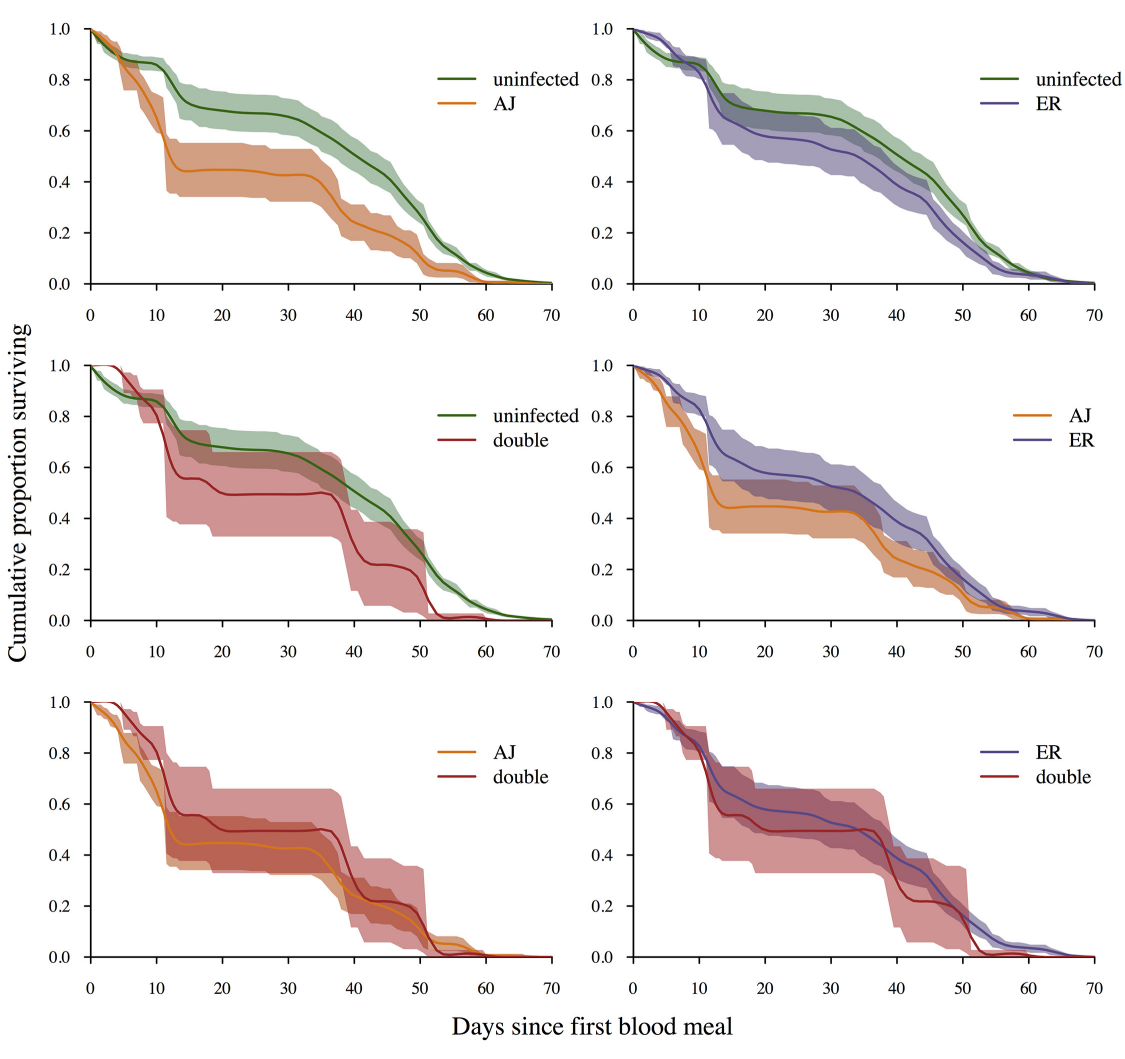
Infection status and vector survival. Survival curves depending on infection status at death (determined by PCR of mosquito cadavers). Lines are a spline fitted to the mean survival curve for mosquitoes with that infection status from between 6 (double infections) and 21 (uninfected) replicate cages. Shaded areas show the standard error of the mean. Mosquitoes infected with AJ had significantly lower survival rates than control mosquitoes (X^**2**^ = 5.58, p = 0.02). All other pairwise comparisons were non-significant (p>0.1). For full analysis see results text and Table F in S2 Text.

There was a significant effect of infection status on mosquito survival (4 level factor; uninfected, AJ infection, ER infection, double infection; Χ^2^_3,891_ = 9.53, p = 0.024). However the only significant pairwise comparison was between uninfected mosquitoes and those infected with AJ alone (AJ vs. uninfected: Χ^2^_1,673_ = 6.5, p = 0.01; ER vs. uninfected: Χ^2^_1,810_ = 1.05, p = 0.31; AJ vs. ER: Χ^2^_1,253_ = 0.24, p = 0.62; Double infection vs. uninfected: Χ^2^_1,638_ = 0.002, p = 0.99; Double infection vs. AJ: Χ^2^_1,81_ = 0.15, p = 0.70; Double infection vs. ER: Χ^2^_1,218_ = 0.002, p = 0.97; Fig 6; Table F in S2 Text), and so we conclude that while there was some evidence of clone differences in virulence, there was no evidence that double infections had a greater virulence to the mosquito than single infections (Fig 6). While this initially seems surprising, given that double infections contained more parasites than single infections, it is likely that all the densities within our experiment where low enough to not have a detectable impact on vector survival, particularly under laboratory conditions with *ad libitum* access to glucose and water [20–22,40].

## Discussion

So far as we are aware, our experiments provide the first conclusive evidence that mosquitoes are capable of accumulating multiple infections over successive blood meals. We found that they are (Fig 1), and furthermore that the presence of parasites from a previous infection facilitates both the establishment and density of subsequent malaria parasite infections (Fig 3, Fig 5) without negatively impacting the replication of the primary infection (Fig 2) or mosquito survival (Fig 6).

Facilitation of establishment and density of secondary infections contrasts with the competitive suppression seen during mixed strain infections in the vertebrate host [9,41]. Previous studies have shown negative density dependence in the production of sporozoites by oocysts, presumably due to resource limitation or apparent competition mediated by the vector immune response [20]. However, parasites in our study are unlikely to have reached the threshold for negative density dependence to impact development (estimated at ~200 oocysts [20]). It is possible that the facilitation we observed is because primary infection leads to structural changes in the mosquito midgut making it easier for a second infection to invade, and/or that the vector’s anti-parasite immune response may be depleted or suppressed by the primary infection, thereby leading to lower ookinete mortality. Another interesting possibility is that parasites respond to cues signalling the presence of another genotype and alter their replication schedules, as can apparently occur in vertebrate infections [9,42]. Changes in vector biting behaviour induced by the primary infection [36], or trade-offs between the duration of oocyst development and sporozoite production, may mean that the fitness-maximizing intrinsic incubation period for malaria parasites is different for parasites sharing the vector with parasites from an existing infection. If this were the case, the higher genome counts from secondary infections could be due to parasites speeding up their replication when entering an already infected mosquito, in order to maximise representation in the salivary glands when the mosquito bites new hosts. Further experiments are required in order to determine how the within-vector environment changes with the establishment of a previous infection and why this increases the probability of a new infection and its density. A good first step would be to track the ookinetes invasion and establishment of oocysts, using fluorescently marked parasites within a previously infected mosquito, and therefore determine at which stage facilitation occurs.

At first glance, our discovery that a primary malaria infection facilitates a subsequent infection contrasts with the finding by Rodrigues *et al*. that midgut bacteria introduced into the mosquito haemolympth by invading ookinetes prime the vector immune response, reducing the density of subsequent malaria parasite infections [43]. Several differences in experimental protocols may account for the apparent contradiction. For example, overall oocyst loads in our experiments were close to natural infection densities [27,44,45] and much lower than those of Rodrigues *et al*. (mean ~5 oocysts per midgut in our single infections compared to means of ~15 & ~60 [43]). Perhaps a large number of ookinetes must cross the midgut to generate sufficient bacterial infection to prime a protective anti-*Plasmodium* effect. Alternatively, our challenge infections were four days after our primary infections. Rodrigues *et al*. [43] challenged their mosquitoes 7 and 14 days later; perhaps anti-malaria immunity elicited by bacterial invasion takes a week or more to develop. The elegant experimental protocols of Rodrigues *et al*. were not designed to look at direct interactions between the priming and challenge parasites because they induced early death of primary infections. Some combination of their protocols and ours would make possible the analysis of the outcomes of co-infections initiated further apart in time and at higher parasite densities. We concentrated on infections acquired from successive blood meals because mosquitoes rarely live long enough to transmit infections acquired two or more gonotrophic cycles after the first [35,46,47].

Combined, our results suggest that mosquitoes taking multiple infective bites will disproportionally contribute to onward malaria transmission of individual strains. How often mosquitoes would be expected to take multiple infective feeds in natural transmission settings depends on many other parameters (e.g. biting rate, proportion of infectious hosts, vector survival). Using parameters from Killeen *et al*. [35] we estimate that without facilitation, ~10–41% of infectious vectors would have oocysts originating from more than one feeding cycle and ~8–17% of infectious mosquitoes would have salivary gland sporozoites originating from multiple blood meals (S1 Text). These estimates are lower bounds; with facilitation these proportions could be much higher. They will be even higher if mosquitoes feed on multiple hosts within a gonadotrophic cycle [27–29], if infected mosquitoes are more likely to blood feed [48], and if infected hosts are more attractive to mosquitoes [49], as has been recorded. Our data are in keeping with the observation that mixed species infections in the field appear to be higher in mosquitoes than would be expected from the single constitutive species prevalence’s, or from the prevalence of mixed infections in humans [4]. Additionally, accumulation of infections multiple feeds could partially explain the lower than expected rates of heterozygous oocysts observed in field studies of *P*. *falciparum* [45](as parasites from multiple feeds will not be able to mate).

The controlled experiments reported here are not feasible in natural transmission settings as they require replicate infections in vertebrate hosts with known infection densities, matched time since infection (to control for transmission blocking immunity) and parasite strains which can be tracked by PCR through the mosquito. However, if mosquitoes in the field are accumulating multiple infections over the course of their lives, we predict that older mosquitoes would have a higher prevalence of mixed infections than younger mosquitoes [4]. With tools now available for determining infection diversity [25,45] and rapid estimation of age of field caught mosquitoes [50], this can be tested.

If the facilitation we have demonstrated here occurs in natural transmission settings, there could be significant epidemiological consequences. Control measures reducing prevalence in the vertebrate host, and therefore reducing the likelihood of mosquitoes taking multiple infective feeds, could disproportionally reduce transmission of individual strains – for example of drug resistant parasites. By increasing the proportion of infectious mosquitoes with mixed strain infections it is also likely that the facilitation reported here will increase the rates of mixed infections in vertebrate hosts which could have implications for infection virulence and the spread of resistant strains [1,51].

More generally, our results point to contrasting effects of mixed strain infections during the malaria lifecycle – while different parasite strains competitively suppress each other in the vertebrate host [6,9,52,53], we have found that they facilitate each other in the mosquito. The potential epidemiological and evolutionary consequences of this antagonism and synergy could be investigated using mathematical models of malaria populations.

## Methods

### Parasites, hosts and vectors

The two wild type *Plasmodium chabaudi* parasite strains (AJ and ER) used here were originally collected from thicket rats (*Thamnomys rutilans*) in the Congo [54], maintained as part of the WHO Registry of Standard Malaria Parasites (The University of Edinburgh) before transportation to Penn State University where they are stored in liquid nitrogen.

Mice in our experiments were 6–10 week old female C57Bl/6 kept on a 12:12 L:D cycle. The mice were fed on Laboratory Rodent Diet 5001 (LabDiet; PMI Nutrition International, Brentwood, MO, USA) and received 0.05% PABA-supplemented drinking water to enhance parasite growth [55]. Infections were established via intraperitoneal (IP) injection with 5x10^5^ parasites. For each transmission, double the number of mice needed were infected 14, 15 or 16 days prior to mosquito bloodmeal. On the day of transmission gametocytemia (proportion of red blood cells containing gametocytes taken from thin blood smears) and red blood cell density (from 2 μL of blood examined by Flow Cytometry, Beckman Coulter Counter; see [56]) was used to calculate the gametocyte density per μL of blood. The mice with infections containing the highest density of gametocytes were selected and anaesthetized with a 5μL IP injection of Ketamine (100 mg/kg) and Xylazine (10 mg/kg) and placed on top of individual mosquito cages for 30 minutes. One mouse was used per feed per cage (experiment 1: 12 mice used for 6 cages; experiment 2: 42 mice used for 21 cages; see Table 1 for treatment groups). As each cage was fed on a different mouse, the density of transmission stages in the blood of each mouse was compared across treatment groups within each experiment, confirming that focal gametocyte densities did not significantly differ (AJ in experiment 1: F_1,4_ = 2.22, p = 0.21; AJ in experiment 2: F_1,4_ = 0.05, p = 0.84; ER in experiment 2: F_1,4_ = 0.71, P = 0.44; see Table A in S2 Text for gametocyte densities in each of the relevant pairwise comparisons). In order to maximise power without increasing the number of animals used, mosquitoes from the cages receiving two infective feeds were used to examine both the effect double infections on both the first and second infection to establish (see Table 1).

*Anopheles stephensi* larvae were reared under standard insectary conditions at 26°C, 85% humidity and a 12L:12D photo-period. Eggs were placed in plastic trays (25 cm × 25 cm × 7 cm) filled with 1.5 L of distilled water. To reduce variation in adult size at emergence, larvae were reared at a fixed density of 400 per tray. Larvae were fed on ground *TetraFin* fish flakes and from 10–11 days after egg hatch, pupae were collected daily and placed in emergence cages. The adults that emerged were fed *ad libitum* on a 10% glucose solution supplemented with 0.05% paraaminobenzoic acid (PABA). Adult female mosquitoes between 3 and 5 days old were equally distributed across all experimental cages with 100–120 female mosquitoes per cage. Experimental cages were given *Ad lib* access to 10% glucose solution supplemented with 0.05% paraaminobenzoic acid (PABA) apart from in the 24 hours prior to feeding on mice where they were deprived of glucose to increase propensity to blood feed. After both blood-feeds, any visibly unfed females were removed and discarded and mosquitoes were provided with bowls for oviposition. Sample sizes in Table 1 reflect the number of mosquitoes that took full bloodmeals on both occasions they were offered a host.

### Measuring infection status and intensity

In order to ensure densities were comparable our focal infections were always assessed after 7 days. This means that when we were testing for an impact on the first infection mosquitoes were dissected at experimental day 7 and when we were testing for an impact on the second infection mosquitoes were dissected at experimental day 11 (7 days after the second bloodmeal on experimental day 4). To determine infection status and density ~30 mosquitoes per cage were removed, killed with chloroform and dissected. Midguts were examined for oocyst presence and intensity and infected guts were then placed individually into 30 μL of chilled PBS within 1.5 mL microtubes. Tubes were maintained on ice prior to storage at -80°C. DNA was extracted from individual mosquito midguts using the E.Z.N.A MicroElute Genomic DNA kit (Omega Bio-Tek) as per manufacturer’s instructions, eluted in a total volume 20 μL and stored at -80°C. Clone specific genome numbers were determined by PCR following the methods in [57].

### The effect of infection status on survival

Cages were checked for dead mosquitoes twice daily until all mosquitoes had died (72 days after receiving their first blood meal). Mosquito cadavers were stored individually in 1.5mL microtubes and immediately frozen at -20°C for short-term storage before being moved to -80°C within two weeks. Parasite DNA was extracted for the mosquito cadavers and the presence and genome count for each strain was quantified using the same methodology as for dissected midguts except for the addition of 2.5μL of BSA per reaction well prior to PCR analysis (10mg/mL Bovine Serum Albumin, New England BioLabs Inc.). BSA was used as pigment found in the eyes of insects has previously been shown to inhibit DNA amplification [58]. A pilot study confirmed previous studies [59], showing BSA was successful at preventing this inhibition. Infection prevalence in dead mosquitoes from each cage strongly correlated with prevalence from dissected mosquitoes confirming our ability to reliably detect parasite infection through this method (R^2^ = 0.99 for AJ; R^2^ = 0.96 for ER prevalence; R^2^ = 0.95 for the mean number of strains per mosquito; S3 Fig).

### Data analysis

All analysis was performed using R version 3.0.2 (R core team (2013) http://www.R-project.org). Gametocyte densities in the mice used for transmission were calculated by multiplying the gametocytemia by the red blood cell density and were log10 transformed and analyzed using general linear models. The proportion of mosquitoes infected with the focal strain for each group was analyzed using generalized mixed effect models (glmer) with a binomial error structure and cage fitted as a random effect (lme4. R package version 1.0–6). For analysis of infection density within the mosquito, only infected mosquitoes were included and host gametocyte density was fitted as a random effect in models. Oocyst densities were analysed using glmer with a poisson error structure and sporozoite densities were log10 transformed and analysed using lmer models. Survival analysis was performed using Cox proportional hazard mixed effect models (Terry Therneau (2012) coxme: Mixed Effects Cox Models. R package version 2.2–3) with experimental cage fitted as a random effect and infection status, estimated total red blood cells in bloodmeals and the mean oocyst density from mosquitoes dissected from the same cage fitted as fixed effects. Total red blood cell density in bloodmeals was estimated from red blood cell densities in the two mice each cage fed on (one per feed) and was included to account for any variation in the quality of bloodmeals received. For all analyses we followed model simplification by sequentially dropping the least significant term and comparing the change in deviance with and without the term to Chi-square distributions until the minimum adequate model was reached. Full details of statistical models can be found in S2 Text and data are deposited in the Dryad repository: (doi:10.5061/dryad.8nr13) [60].

### Ethics statement

This study was carried out in strict accordance with the recommendations in the Guide for the Care and Use of Laboratory Animals of the National Institutes of Health. The protocol was approved by the Animal Care and Use Committee of the Pennsylvania State University (Permit Number: 35790).

## Supporting Information

S1 Text Predicted contribution of mosquitoes infected or infectious with parasites from multiple feeds to transmission in the absence of facilitation. Methods A: General details of the explicit feeding cycle model used to simulate survival and infection. Figure A: Predicted contribution of mosquitoes infected or infectious with parasites from multiple feeds for each of four transmission settings. Table A: Summary of the epidemiological characteristics of the four transmission settings used in simulations and example simulation results. Figure B: Predicted contribution of mosquitoes infected or infectious with parasites from multiple feeds with a cut-off for survival at five post infection feeding cycles. Methods B: R code for simulations.
(DOCX)

S2 Text Extra details for infection parameters and statistical analyses. Table A: Comparison of gametocyte densities in the mice used for each pairwise comparison of infections. Table B: Full details for “The affect of secondary infection on replication of primary infection” analysis. Table C: Full details for the “The effect of previous infection on probability of secondary infection” analysis. Table D: Full details for “Oocyst density in single and double infections” analysis. Table E: Full details for “The effect of a previous infection on the replication of subsequent infection” analysis. Table F: Full details for “The effect of infection status on vector survival” analysis.
(DOCX)

S1 Fig Ability to detect oocyst infections from first feed at day 7 and day 8. Data from experiment 1. Mosquitoes were given an infected bloodmeal on day 0 (with clone AJ), an uninfected bloodmeal on day 4. Midguts where then removed examined for oocysts and PCR’d for the presence of AJ genomes at day 7 and day 11. There was no significant difference in the prevalence of infection at these two time points (Χ^2^_1,4_ = 0.16, p = 0.69) indicating that we could reliably detect the presence of an infection received on day 0 at day 11 (the appropriate time-point for examining infections received on day 4). Means are based on dissection of 90 mosquitoes across 3 replicate cages for each time point. Bars show the standard error of the mean.
(TIFF)

S2 Fig Relationship between genome counts of AJ and ER within individual mosquitoes. Density of ER and or AJ in dissected mosquitoes that became infected with parasites from both bloodmeals. Each point represents a single mosquito. ER density ~ AJ density; Χ^2^_1,8_ = 0.002, p = 0.97.
(TIFF)

S3 Fig Correlation between prevalence in dead and dissected mosquitoes. Each point shows a cage mean for prevalence/number of strains per mosquito determined though PCR of dead mosquitoes throughout the experiment or from dissection and PCR of midguts 7 days after an infective feed. The black line shows a 1 to 1 correlation for reference and the R squared values for each relationship are displayed on the graph.
(TIFF)
