# Supplementary material for: Existing Infection Facilitates Establishment and Density of Malaria Parasites in Their Mosquito Vector
Source: PLoS Pathog. 2015 Jul 16;11(7):e1005003. doi: 10.1371/journal.ppat.1005003 (PMC4504473; doi:10.1371/journal.ppat.1005003)
Supplement: S1 Text — Methods A: General details of the explicit feeding cycle model used to simulate survival and infection. Figure A: Predicted contribution of mosquitoes infected or infectious with parasites from multiple feeds for each of four transmission settings. Table A: Summary of the epidemiological characteristics of the four transmission settings used in simulations and example simulation results. Figure B: Predicted contribution of mosquitoes infected or infectious with parasites from multiple feeds with a cut-off for survival at five post infection feeding cycles. Methods B: R code for simulations. (DOCX) [file ppat.1005003.s001.docx]

**S1: Methods for simulation of proportion of infectious bites contributed by mosquitoes infected or infectious with parasites from multiple feeds**

**Methods A**

Using an explicit feeding cycle model [1,2] we simulated survival and infection for 100000 mosquitoes until death (or until a cut-off). Simulations were repeated across 4 transmission settings taking fixed values for the following parameters taken from Killeen *et al.* 2000 [3].

- Extrinsic incubation period (EIP): The length of time (in days) for the parasite to reach the salivary glands.
- Survival (pf): Probability of surviving to the next feeding cycle
- Infection probability (k): probability of becoming infected per bite on a human host. As we assume mosquitoes bite one human per feeding cycle this is also the probability of becoming infected per feeding cycle.
- Length of feeding cycle (f): Number of days between blood feeding.
- First infectious feed (F): First feed in which a mosquito could be infected.

Additionally we set a parameter (MF) to provide a maximum number of feeding cycles and to prevent an unrealistically long tail of surviving mosquitoes as we were assuming age independent exponential mortality at a rate of pf per gonotrophic cycle. In our initial simulation MF was set at 10 post infectious gonotrophic cycles to fit with maximum rates from mark-recapture studies [4] and survival in our experiments. Simmulations were additionally ran with MF set to 5 (Figure S1.2). Our simulation assumes each mosquito bites one human host per feeding cycle, doesn’t skip feeds and that the probability of picking up secondary infections is independent of the first. Mosquitoes have a fixed probability of death each feeding cycle (pf) and if they become infected there is a fixed lag (EIP) before they are they become infective. Mosquitoes do not clear infections. For each feeding cycle we calculated, the total number of infectious bites and of these how many came from a mosquitos which were multiply infected (had oocysts originating from >1 feed) or multiply infective (had sporozoites originating from >1 feed).

**Figure A**: **Predicted contribution of mosquitoes infected or infectious with parasites from multiple feeds to transmission in the absence of facilitation.**  Infectious mosquitoes with parasites originating from a single bloodmeal are shown in blue. Infectious mosquitoes with oocysts originating from two or more bloodmeals are shown in orange. Mosquitoes with salivary gland sporozoites originating from two or more bloodmeals are shown in red. The left hand panels show the relative contribution of each infection type to the pool of infectious mosquitoes over time. The right hand panels show the overall contribution (area under the curve) to transmission of mosquitoes with oocysts (multiply infected) or sporozoites (multiply infective) originating from more than one feed.

**Table A:** Summary of the epidemiological characteristics of the four transmission settings and example simulation results.

| Fixed parameters (from Killeen *et al.* 2000) | | | | | | Simulation results (summed across feeding cycles) | | | | |
| --- | --- | --- | --- | --- | --- | --- | --- | --- | --- | --- |
| Location | EIP | pf | k | f | F | Starting #  mosquitoes | Total # infectious bites | Bites from mosquitoes with 1 infection | Bites from mosquitoes with >1 infection (oocysts) | Bites from mosquitoes with >1 infection (sporozoites) |
| Butelgu, P.N.G | 9 | 0.58 | 0.074 | 3.7 | 2.3 | 1,000,000 | 128581 | 77.8% | 22.2% | 9.3% |
| Kankiya, Nigeria | 10.3 | 0.83 | 0.024 | 3 | 3.4 | 1,000,000 | 372598 | 85.2% | 14.8% | 8.3% |
| Kanduna, Nigeria | 11.6 | 0.81 | 0.055 | 2 | 5.8 | 1,000,000 | 321582 | 59.3% | 40.7% | 16.5% |
| Namawala, Tanzania | 11 | 0.62 | 0.018 | 2.7 | 4.3 | 1,000,000 | 18186 | 90.0% | 10.0% | 2.9% |

**Figure B**: **Reducing the maximum survival limited to 5 post infection feeding cycles:** Simulations were rerun as before but with MF set to 5. This led to slight reductions in the number of mosquitoes with sporozoites from multiple feeds.

**Methods B: R code for simulations**

Simulations were performed in R (R Core Team (2013). R: http://www.R-project.org/.) Example R code for one of the locations (Kanduna, Nigeria) is included below.

## Kanduna, Nigeria

EIP=11.6 # Extrinsic incubation period

pf=0.81 # probability of surviviving each feeding cycle

k=0.055 # probability of becoming infected per human bite

f=2 # number days between feeds

F=round(5.8) # first feed they could be infectious\

MF=10 # cut off maximum number of feeding cycles

n_mosies<-1000000 #starting number of mosquitoes

# create three matrixes for survival (s), infected (i) and infective (inf).

s<-matrix(0,nrow=n_mosies,ncol=(18+F))

i<-matrix(0,nrow=n_mosies,ncol=(18+F))

inf<-matrix(0,nrow=n_mosies,ncol=(18+F))

s[,1]<-1 # assume they survive to take first bite so 1^st^ column filled with 1’s

i[,1]<-rbinom(n_mosies,1,k) # Probability of becoming infected on 1^st^ bite

inf[,1:F]<-0 # No mosquitoes can have sporozoites till F

for (f in 2:(MF+F)) {

s[,f]<-ifelse(s[,f-1]>0,rbinom(n_mosies,1,pf),0)

i[,f]<-(rbinom(n_mosies,1,k))+i[,f-1]

}

for (f in (F+1):(MF+F)) {

inf[,f]<-i[,(f-F)]

}

feed<-c(rep(c((F+1):(MF+F)),each=n_mosies))

survived<-c(s[,7],s[,8],s[,9],s[,10],s[,11],s[,12],s[,13],s[,14],s[,15],s[,16])

infected<-c(i[,7],i[,8],i[,9],i[,10],i[,11],i[,12],i[,13],i[,14],i[,15],i[,16])

infective<-c(inf[,7],inf[,8],inf[,9],inf[,10],inf[,11],inf[,12],inf[,13],inf[,14],inf[,15],inf[,16])

mult.infective<-ifelse(infective>1,1,0)

mult.infected<-ifelse(infected>1,1,0)

# make a data frame of simulation results and then subset to only include alive and infectious mosquitoes

data<-data.frame(feed,survived,infected,infective,mult.infected,mult.infective)

alive.data<-data[which(data$survived==1),]

infective.data<-alive.data[which(alive.data$infective>0),]

# aggregate data by infection status and gonotrophic cycle number

data.means<aggregate(infective.data$survived,by=list(infective.data$mult.infected,infective.data$mult.infective,infective.data$feed),sum)

data.means<rename(data.means,c("Group.1"="multiple.infected","Group.2"="multiple.infective","Group.3"="feed","x"="n"))

total.n<-sum(data.means$n) # total number of infectious bites

data.means$rel.n<-data.means$n/total.n

feed.totals<-aggregate(infective.data$survived,by=list(infective.data$feed),sum)

rel.per.feed<-feed.totals$x/total.n

m.infected.per.feed<rel.per.feed(data.means$rel.n[data.means$multiple.infected==0&data.means$multiple.infective==0])

m.infective.per.feed<c(0,data.means$rel.n[data.means$multiple.infected==1&data.means$multiple.infective==1])

days<-c((F+1):(F+MF))

time.graph.kanduna<-data.frame(days,rel.per.feed,m.infected.per.feed,m.infective.per.feed)

total.n.kanduna<-sum(data.means$n)

single.kanduna<sum(data.means$n[data.means$multiple.infected==0&data.means$multiple.infective==0])

m.infected.kanduna<sum(data.means$n[data.means$multiple.infected==1&data.means$multiple.infective==0])

m.infective.kanduna<sum(data.means$n[data.means$multiple.infected==1&data.means$multiple.infective==1])

**References**

1. Cator LJ, Lynch PA, Thomas MB, Read AF (2014) Alterations in mosquito behaviour by malaria parasites: potential impact on force of infection. Malar J 13:164.
2. Lynch PA, Grimm U, Thomas MB, Read AF (2012) Prospective malaria control using entomopathogenic fungi: comparative evaluation of impact on transmission and selection for resistance. Malar J 11: 383
3. Killeen GF, McKenzie FE, Foy BD, Schieffelin C, Billingsley PF, *et al.* (2000) A simplified model for predicting malaria entomologic inoculation rates based on entomologic and parasitologic parameters relevant to control. Am J Trop Med Hyg 62: 535-544.
4. Boyd MF (1949) Epidemiology: Factors related to the definitive host. In: Boyd MF, editor. Malariology. A comprehensive survey of all aspects of this group of diseases from a global standpoint. Volume 1. Philadelphia: W. B. Saunders Company, Vol. 1. pp. 609–697.
