# Supplementary material for: Existing Infection Facilitates Establishment and Density of Malaria Parasites in Their Mosquito Vector
Source: PLoS Pathog. 2015 Jul 16;11(7):e1005003. doi: 10.1371/journal.ppat.1005003 (PMC4504473; doi:10.1371/journal.ppat.1005003)
Supplement: S2 Text — Table A: Comparison of gametocyte densities in the mice used for each pairwise comparison of infections. Table B: Full details for “The affect of secondary infection on replication of primary infection” analysis. Table C: Full details for the “The effect of previous infection on probability of secondary infection” analysis. Table D: Full details for “Oocyst density in single and double infections” analysis. Table E: Full details for “The effect of a previous infection on the replication of subsequent infection” analysis. Table F: Full details for “The effect of infection status on vector survival” analysis. (DOCX) [file ppat.1005003.s002.docx]

**S3: Extra details for statistical analyses**

| **Table A: Comparison of densities in the mice used for each pairwise comparison between cages**. Means and standard errors refer to the density of gametocytes per μL of mouse blood at the time of blood meal. There were no significant differences in the gametocyte densities fed to any of our comparison groups but gametocyte density was included in all maximal models in order to control for minor variability. | | | | | | | |
| --- | --- | --- | --- | --- | --- | --- | --- |
| **Gametocyte density in focal infections (per μL of blood)** | | | | | | | |
| **Focal infection in 1st feed** | | | | | | | |
| Experiment | Focal | Subsequent feed | Mean | ±SE | df, resid | F | p |
| 1 | AJ | Control | 5.04 x 10^6^ | 0.33 x 10^6^ | 1,4 | 2.04 | 0.23 |
|  |  | ER | 9.08 x 10^6^ | 2.68 x 10^6^ |  |  |  |
| 2 | AJ | Control | 5.28 x 10^6^ | 1.23 x 10^6^ | 1,4 | 1.05 | 0.36 |
|  |  | ER | 7.83 x 10^6^ | 2.12 x 10^6^ |  |  |  |
|  | ER | Control | 2.66 x 10^7^ | 1.55 x 10^6^ | 1,4 | 0.07 | 0.81 |
|  |  | AJ | 2.72 x 10^7^ | 1.52 x 10^6^ |  |  |  |
| **Focal infection in 2nd feed** | | |  |  |  |  |  |
| Experiment | Focal | Previous feed | Mean | ±SE | df, resid | F | p |
| 1 | AJ | Control | 7.18 x 10^6^ | 1.14 x 10^6^ | 1,4 | 0.95 | 0.39 |
|  |  | ER | 5.70 x 10^6^ | 0.99 x 10^6^ |  |  |  |
| 2 | AJ | Control | 5.28 x 10^6^ | 2.12 x 10^6^ | 1,4 | 0.08 | 0.79 |
|  |  | ER | 5.94 x 10^6^ | 2.10 x 10^6^ |  |  |  |
|  | ER | Control | 1.68 x 10^7^ | 1.36 x 10^6^ | 1,4 | 0.68 | 0.46 |
|  |  | AJ | 1.93 x 10^7^ | 2.59 x 10^6^ |  |  |  |
| **Red blood cell density for focal infections (per μL of blood)** | | | | | | | |
| **Focal infection in 1st feed** | | | | | | | |
| Experiment | Focal | Subsequent feed | Mean | ±SE | df, resid | F | p |
| 1 | AJ | Control | 5.17 x 10^9^ | 0.59 x 10^9^ | 1,4 | 0.15 | 0.72 |
|  |  | ER | 5.37 x 10^9^ | 0.31 x 10^9^ |  |  |  |
| 2 | AJ | Control | 3.89 x 10^9^ | 0.23 x 10^9^ | 1,4 | 0.07 | 0.81 |
|  |  | ER | 4.31 x 10^9^ | 0.98 x 10^9^ |  |  |  |
|  | ER | Control | 2.34 x 10^9^ | 0.12 x 10^9^ | 1,4 | 0.94 | 0.39 |
|  |  | AJ | 2.7 x 10^9^ | 0.34 x 10^9^ |  |  |  |
| **Focal infection in 2nd feed** | | | | | | | |
| Experiment | Focal | Previous feed | Mean | ±SE | df, resid | F | p |
| 1 | AJ | Control | 6.07 x 10^9^ | 0.42 x 10^9^ | 1,4 | 1.82 | 0.25 |
|  |  | ER | 4.56 x 10^9^ | 1.10 x 10^9^ |  |  |  |
| 2 | AJ | Control | 3.93 x 10^9^ | 0.23 x 10^9^ | 1,4 | 1.30 | 0.32 |
|  |  | ER | 3.53 x 10^9^ | 0.27 x 10^9^ |  |  |  |
|  | ER | Control | 3.48 x 10^9^ | 0.07 x 10^9^ | 1,4 | 3.75 | 0.13 |
|  |  | AJ | 3.31 x 10^9^ | 0.06 x 10^9^ |  |  |  |

| **Table B: The effect of secondary infection on the density of the primary infection.** Data from infected mosquitoes in experiment 2. Focal strain refers to the strain received in the first feed. | | | | | | | | | | | | | |
| --- | --- | --- | --- | --- | --- | --- | --- | --- | --- | --- | --- | --- | --- |
| Summary statistics | | | | | | | | | | | | | |
| Focal strain | Second feed | | | | | | n (mosquitoes, cages) |  | Mean focal genomes | | | (± SE) | |
| AJ | Control | | | | | | 44, 3 |  | 11428 | | | 2842 | |
|  | Infective (ER) | | | | | | 32,3 |  | 10318 | | | 2305 | |
| ER | Control | | | | | | 58, 3 |  | 438651 | | | 86479 | |
|  | Infective (AJ) | | | | | | 77,3 |  | 550164 | | | 123394 | |
| Analysis - Focal Infection Genomes (log10) | | | | | | | | | | | | | |
|  | | | | | | | | | | d.f for term | χ**^2^** | | p |
| Model | | lmer | | | | | | | |  |  | |  |
| Fixed effects | | Focal strain (2 level factor: AJ, ER) | | | | | | | | 1 | 21.13 | | <0.001 |
|  | | Status of 2nd feed (2 level factor: Control, Infective) | | | | | | | | 1 | 0.09 | | 0.77 |
|  | | Status of 2^nd^ feed * focal strain | | | | | | | | 1 | 0.20 | | 0.66 |
|  | | Red blood cell density of second feed | | | | | | | | 1 | 1.013 | | 0.31 |
| Random effect | | 1\| Gametocyte density of focal infection | | | | | | | |  |  | |  |
| **Split by whether second infection established** | | | | | | | | | | | | | |
| Summary statistics | | | | | | | | | | | | | |
| Focal strain | | Second feed | | | | n (mosquitoes, cages) | | | Mean focal genomes | | | (± SE) | |
| AJ | | Control | | | | 44, 3 | | | 11428 | | | 2842 | |
|  | | Exposure (no infection) | | | | 10, 3 | | | 11174 | | | 4670 | |
|  | | Infection with ER | | | | 22, 3 | | | 9928 | | | 2670 | |
| ER | | ER alone | | | | 58, 3 | | | 438651 | | | 86479 | |
|  | | Exposure (no infection) | | | | 68, 3 | | | 272604 | | | 46119 | |
|  | | Infection with ER | | | | 9, 3 | | | 2701250 | | | 650610 | |
| Analysis - Focal Infection Genomes (log10) | | | | | | | | | | | | | |
| Model | | | lmer | | | | | | |  |  | |  |
| Fixed effects | | | Focal strain (2 level factor: AJ, ER) | | | | | | | 1 |  | |  |
|  | | | Status of 2nd feed (3 level factor: Control, Exposed, Infected) | | | | | | | 2 |  | |  |
|  | | | Status of 2^nd^ feed * focal strain | | | | | | | 2 | 14.79 | | <0.001 |
| Random effect | | | 1\| Gametocyte density of focal infection | | | | | | |  |  | |  |
| *Tukey pairwise comparisons* | | | | | | | | | |  | *z* | | p |
| AJ (control) | | | | vs. | AJ (exposed) | | | | |  | 0.08 | | 0.99 |
| AJ (control) | | | | vs. | AJ (Infected with ER) | | | | |  | 0.24 | | 0.99 |
| AJ (exposed) | | | | vs. | AJ (Infected with ER) | | | | |  | 0.45 | | 0.99 |
|  | | | |  |  | | | | |  |  | |  |
| ER (Control) | | | | vs. | ER (exposed) | | | | |  | 0.45 | | 0.99 |
| ER (Control) | | | | vs. | ER (Infected with AJ) | | | | |  | 3.49 | | 0.01 |
| ER (exposed) | | | | vs. | ER (Infected with AJ) | | | | |  | 4.7 | | <0.005 |
|  | | | |  |  | | | | |  |  | |  |
| ER (control) | | | | vs. | AJ (control) | | | | |  | 4.09 | | <0.001 |

| **Table C: The effect of previous infection on probability of secondary infection.** Focal strain refers to the strain received in the second feed. | | | | | | | | |
| --- | --- | --- | --- | --- | --- | --- | --- | --- |
| Summary statistics | | | | | | | | |
|  | Focal strain | | Infection status | n (mosquitoes, cages) | Mean % infected | | | (± SE) |
| Experiment 1 | AJ | | Control | 92, 3 | 0.152 | | | 0.038 |
|  |  | | Exposed | 53, 3 | 0.019 | | | 0.018 |
|  |  | | Infected | 24, 3 | 0.500 | | | 0.104 |
| Experiment 2 | AJ | | Control | 90, 3 | 0.033 | | | 0.019 |
|  |  | | Exposed | 23, 3 | 0.087 | | | 0.060 |
|  |  | | Infected | 68, 3 | 0.250 | | | 0.053 |
|  | ER | | Control | 86, 3 | 0.326 | | | 0.051 |
|  |  | | Exposed | 65, 3 | 0.369 | | | 0.060 |
|  |  | | Infected | 23, 3 | 0.783 | | | 0.088 |
| Analysis | | | | | | | | |
| **Experiment 1** | | | | | | | | |
|  | | | | | d.f for term | χ**^2^** | p | |
| Model 1.1 | | glmer with binomial error structure | | |  |  |  | |
| Fixed effects | | Infection status (3 level factor: control, exposed, infected) | | | 2 | 26.89 | <0.0001 | |
|  | | Red blood cell density in previous feed | | | 1 | 0.01 | 0.99 | |
| Random effects | | 1\|cage |  | |  |  |  | |
|  | | | | |  |  |  | |
| Model 1.2 | | glmer with binomial error structure | | |  |  |  | |
| Fixed effects | | Infection status (2 level factor: Uninfected, infected) | | | 1 | 21.38 | <0.0001 | |
| Random effects | | 1\|cage |  | |  |  |  | |
| Model 1.1 vs. Model 1.2 | | | | | 1 | 2.04 | 0.10 | |
| **Experiment 2** | | |  | |  |  |  | |
|  | | | | | d.f for term | χ**^2^** | p | |
| Model 2.1 | | glmer with binomial error structure | | |  |  |  | |
| Fixed effects | | Infection status (3 level factor: control, exposed, infected) | | | 2 | 9.14 | 0.009 | |
|  | | Focal parasite (2 level factor: AJ, ER) | | | 1 | 8.30 | 0.004 | |
|  | | Infection status * Focal parasite | | | 2 | 0.44 | 0.80 | |
|  | | Red blood cell density in previous feed | | | 1 | 0.10 | 0.75 | |
| Random effects | | 1\|cage | | |  |  |  | |
| Model simplification for pairwise comparisons | | | | |  |  |  | |
| Model 2.2 | | glmer with binomial error structure | | |  |  |  | |
| Fixed effects | | Infection status (2 level factor: Uninfected, infected) | | | 1 | 7.09 | 0.008 | |
|  | | Focal parasite (2 level factor: AJ, ER) | | | 1 | 7.83 | 0.005 | |
| Random effects | | 1\|cage | | |  |  |  | |
| Model 1.1 vs. Model 1.2 | | | | | 1 | 2.05 | 0.15 | |

| **Table D: Oocyst density in single and double infections.** Data from infected mosquitoes in experiment 2. Focal strain refers to the strain received in the second feed. | | | | | | | | | | |
| --- | --- | --- | --- | --- | --- | --- | --- | --- | --- | --- |
| Summary statistics | | | | | | | | | | |
|  | Focal strain | | Infection | n (mosquitoes, cages) |  | | Mean | | (± SE) | |
| Oocysts | AJ | | AJ alone | 14, 3 |  | | 5.57 | | 3.15 | |
|  |  | | Double infection | 12, 3 |  | | 7.58 | | 3.96 | |
|  | ER | | ER alone | 28, 3 |  | | 4.68 | | 0.85 | |
|  |  | | Double infection | 42, 3 |  | | 24.07 | | 4.37 | |
| Analysis | | | | | | | | | | |
| **Number of oocysts per mosquito** | | | | | | | | | | |
|  | | | | | | d.f for term | | χ**^2^** | | p |
| Model | | glmer with poisson error structure | | | |  | |  | |  |
| Fixed effects | | Infection status (2 level factor: Single infection, double infection) | | | | 1 | |  | |  |
|  | | Focal strain (2 level factor: AJ, ER) | | | | 1 | |  | |  |
|  | | Infection status * focal strain | | | | 1 | | 60.1 | | <0.005 |
| Random effects | | 1\|cage | | | | 1 | |  | |  |
| Tukey pairwise comparisons | | | | | |  | | z | | p |
| Focal = AJ: | | AJ alone vs. double infection | | | | | | 0.46 | | 0.96 |
| Focal = ER: | | ER alone vs. double infection | | | | | | 4.28 | | <0.001 |
|  | | AJ alone vs. ER alone | | | | | | 0.92 | | 0.78 |

| **Table E: The effect of a previous infection on replication of subsequent infection.** Data from infected mosquitoes in experiment 2. Focal strain refers to the strain received in the second feed. | | | | | | | | | |
| --- | --- | --- | --- | --- | --- | --- | --- | --- | --- |
| Summary statistics | | | | | | | | | |
|  | Focal strain | | Infection | n (mosquitoes, cages) | Focal strain genomes (Mean) | | | (± SE) | |
| Genomes | AJ | | Alone | 14, 3 | 5,477 | | | 1382 | |
|  |  | | In a double infection | 12, 3 | 1,822,136 | | | 1,338,924 | |
|  | ER | | Alone | 28, 3 | 31,771 | | | 6,894 | |
|  |  | | In a double infection | 42, 3 | 175,073 | | | 42,358 | |
| Analysis | | | | | | | | | |
| **Focal Infection Genomes (log10)** | | | | | | d.f for term | χ^2^ | | p |
| Model | | lmer | | | |  |  | |  |
| Fixed effects | | Infection status (2 level factor: Single infection, double infection) | | | | 1 | 8.15 | | 0.004 |
|  | | Gametocyte density for focal infection | | | | 1 | 1.28 | | 0.26 |
|  | | Focal strain (2 level factor: AJ, ER) | | | | 1 | 0.13 | | 0.72 |
| Random effects | | 1\|cage | | | | 1 |  | |  |

| **Table F:** **The effect of infection status on vector survival.** | | | |
| --- | --- | --- | --- |
| Cox proportional hazards models with cage as a random effect | | | |
| Maximal model = coxme(Surv(day,death.status)~infection.status+mean.oocyst+mean.rbc+total.gametocytes+(1\|cage)) | | | |
| Model terms (terms remaining in minimal in bold) | d.f for term | χ**^2^** | p |
| Red blood cell density in blood meals  (mean from two feeds) | 1 | 0.001 | 0.97 |
| Mean oocyst density  (taken from dissection of subset of mosquitoes from cage) | 1 | 0.84 | 0.36 |
| Total gametocytes from feeds  (gametocyte density in 1^st^ feed + gametocyte density in 2^nd^ feed) | 1 | 3.04 | 0.08 |
| **Infection status**  **(factor with 4 levels: Uninfected, AJ, ER, Both)** | **3** | **9.47** | **0.024** |
| **Random effect = cage (factor with 21 levels)** |  |  |  |
| Pairwise comparisons for infection status | | | |
| **Uninfected vs. Infected with AJ alone** | **1** | **5.58** | **0.018** |
| Uninfected vs. Infected with ER alone | 1 | 0.016 | 0.90 |
| Uninfected vs. Infected with AJ+ER | 1 | 2.13 | 0.144 |
| Infected with AJ alone vs. Infected with ER alone | 1 | 1.32 | 0.25 |
| Infected with AJ alone vs. Infected with AJ+ER | 1 | 2.32 | 0.13 |
| Infected with ER alone vs. Infected with AJ+ER | 1 | 0.12 | 0.72 |
