# Supplementary figures and images for: Existing Infection Facilitates Establishment and Density of Malaria Parasites in Their Mosquito Vector
Source: PLoS Pathog. 2015 Jul 16;11(7):e1005003. doi: 10.1371/journal.ppat.1005003 (PMC4504473; doi:10.1371/journal.ppat.1005003)

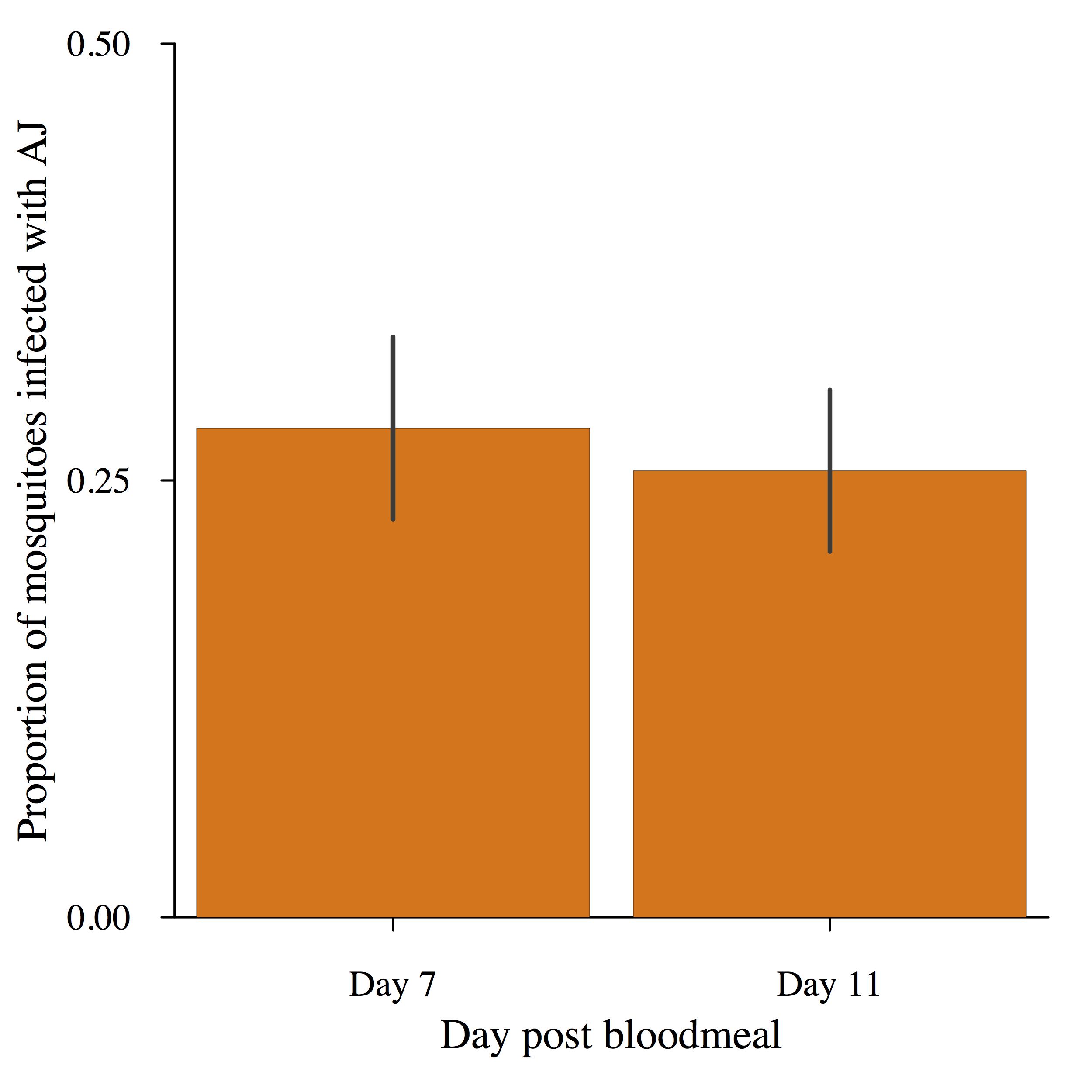

Supplement: S1 Fig — Data from experiment 1. Mosquitoes were given an infected bloodmeal on day 0 (with clone AJ), an uninfected bloodmeal on day 4. Midguts where then removed examined for oocysts and PCR’d for the presence of AJ genomes at day 7 and day 11. There was no significant difference in the prevalence of infection at these two time points (Χ2 1,4 = 0.16, p = 0.69) indicating that we could reliably detect the presence of an infection received on day 0 at day 11 (the appropriate time-point for examining infections received on day 4). Means are based on dissection of 90 mosquitoes across 3 replicate cages for each time point. Bars show the standard error of the mean. (TIFF) [file ppat.1005003.s003.tiff]

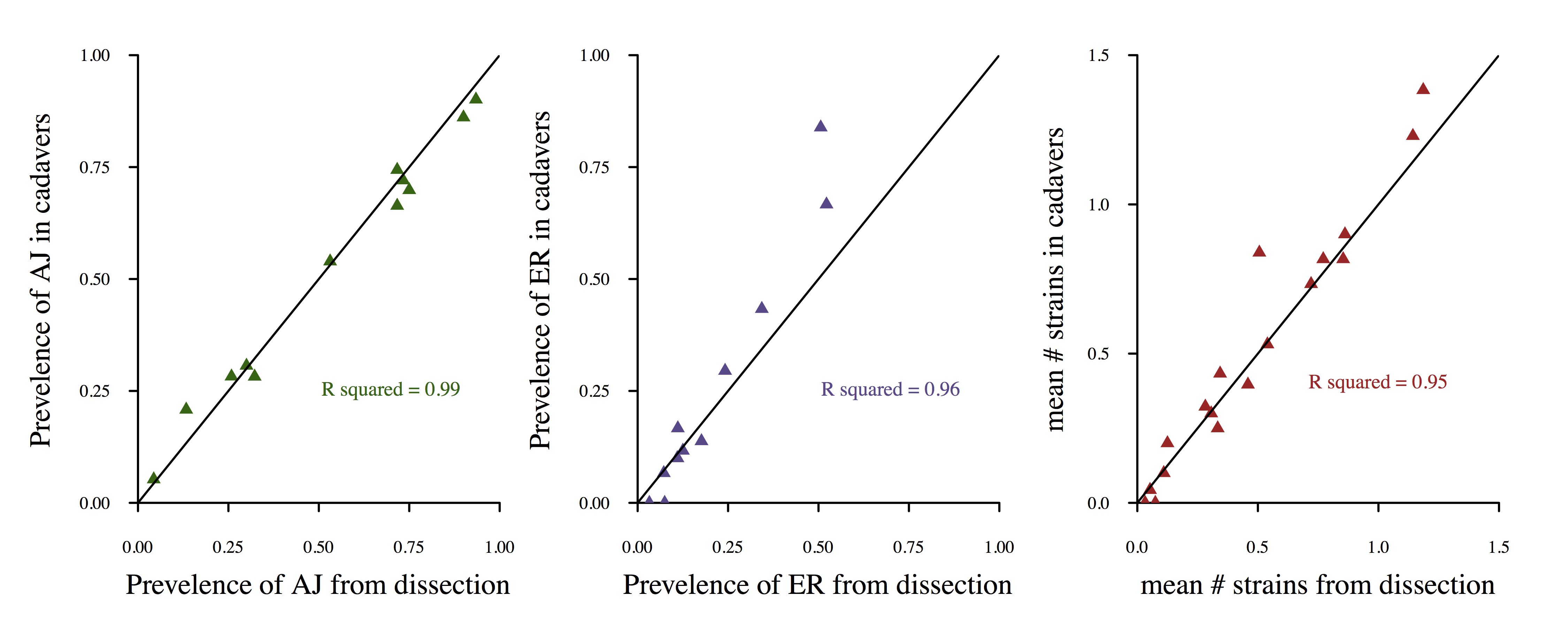

Supplement: S3 Fig — Each point shows a cage mean for prevalence/number of strains per mosquito determined though PCR of dead mosquitoes throughout the experiment or from dissection and PCR of midguts 7 days after an infective feed. The black line shows a 1 to 1 correlation for reference and the R squared values for each relationship are displayed on the graph. (TIFF) [file ppat.1005003.s005.tiff]
